# Supplementary material for: New insights into the mechanisms of plant isotope fractionation from combined analysis of intramolecular 13 C and deuterium abundances in Pinus nigra tree‐ring glucose
Source: New Phytol. 2024 Sep 23;245(3):1000–17. doi: 10.1111/nph.20113 (PMC11711956; doi:10.1111/nph.20113)
Supplement: Supplementary file 1 — Fig. S1 Air vapour pressure deficit of the growing season and March–July precipitation over the period from 1961 to 1995 in the Vienna basin. Notes S1 Materials and Methods (expanded). Notes S2 Hydro‐carbon isotope fractionation from 1961 to 1980. Notes S3 Estimated deuterium fractionation due to shifts of the phosphoglucose isomerase reaction. Table S1 Shapiro–Wilk normality test. Table S2 F and T test. Table S3 Pearson's correlations between Δ i ′ and ε met series of the period from 1983 to 1995. Table S4 Components of variance in Δ i ′ series. Table S5 Pearson's correlation coefficients and associated levels of significance of ε met‐climate relationships for the period from 1983 to 1995. Table S6 Linear regression model of ε met (H1) as function of growing season air vapour pressure deficit and March–July precipitation. Please note: Wiley is not responsible for the content or functionality of any Supporting Information supplied by the authors. Any queries (other than missing material) should be directed to the New Phytologist Central Office. [file NPH-245-1000-s001.pdf]

New Phytologist

***New Phytologist* Supporting Information**

Article title: **New insights into the mechanisms of plant isotope fractionation from combined analysis of intramolecular ^{13}C and deuterium abundances in *Pinus nigra* tree-ring glucose**

Authors: Thomas Wieloch, Meisha Holloway-Phillips, Jun Yu, Totte Niittylä

Article acceptance date: 20 August 2024

The following Supporting Information is available for this article:

Figure S1 Air vapour pressure deficit of the growing season and March to July precipitation over the period from 1961 to 1995 in the Vienna basin.

Notes S1 Materials and Methods (expanded).

Notes S2 Hydro-carbon isotope fractionation from 1961 to 1980.

Notes S3 Estimated deuterium fractionation due to shifts of the phosphoglucose isomerase reaction.

Table S1 Shapiro-Wilk normality test.

Table S2 F and T test.

Table S3 Pearson's correlations between Δ_i' and ε_{met} series of the period from 1983 to 1995.

Table S4 Components of variance in Δ_i' series.

Table S5 Pearson's correlation coefficients and associated levels of significance of ε_{met}-climate relationships for the period from 1983 to 1995.

Table S6 Linear regression model of $\varepsilon_{met\ (H1)}$ as function of growing season air vapour pressure deficit and March to July precipitation.

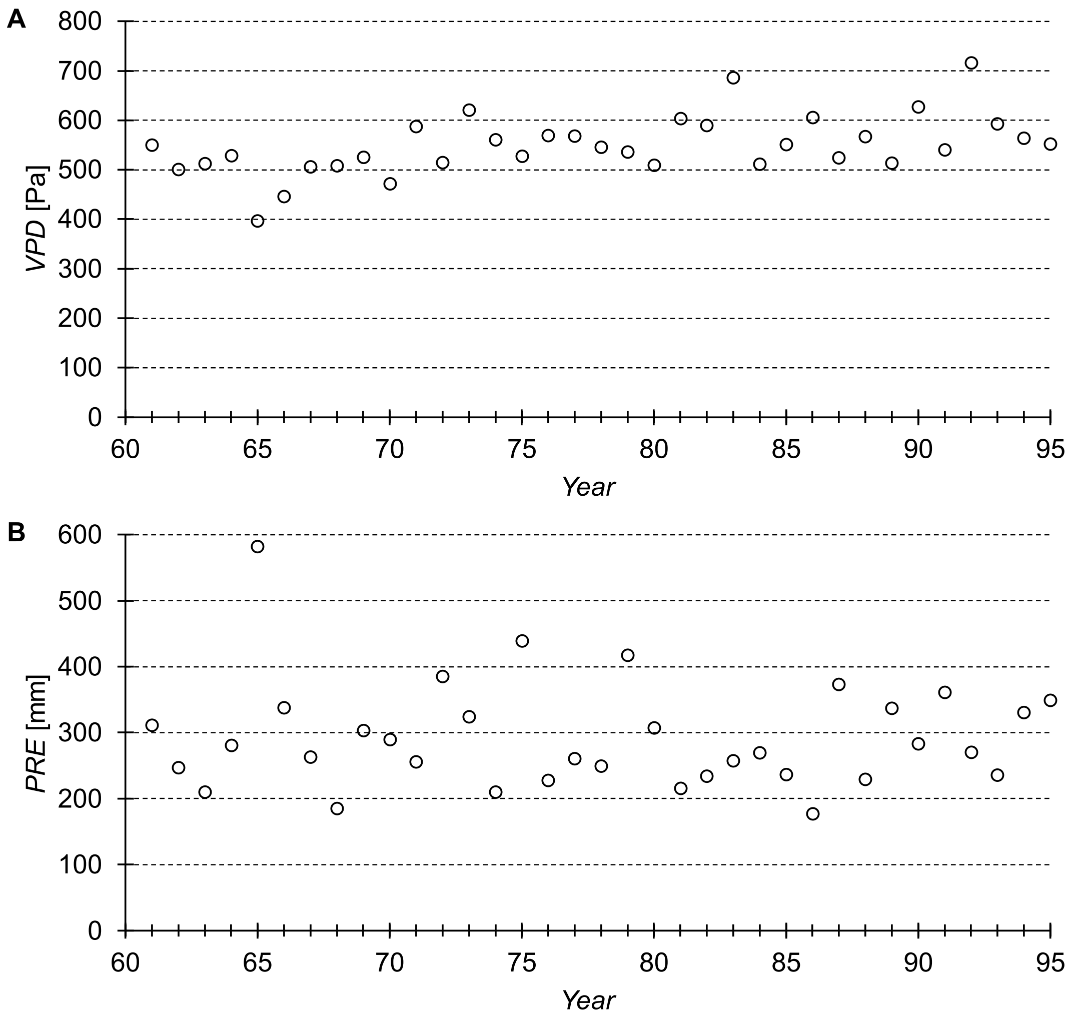

Figure S1 (A) Air vapour pressure deficit (*VPD*) of the growing season and **(B)** March to July precipitation (*PRE*) over the period 1961 to 1995 in the Vienna basin. Raw data were measured at the climate station Hohe Warte (Central Institution for Meteorology and Geodynamics, Vienna, Austria, 48.23° N, 16.35° E, 198 m AMSL, WMO ID: 1103500) (Klein Tank *et al.*, 2002). *VPD* was calculated following published procedures (Abtew & Melesse, 2013).

Notes S1 Materials and Methods (expanded).

The tree-ring samples used here were described previously (Leal *et al.*, 2008). Two tree-ring cores were taken from each of 19 *Pinus nigra* Arnold trees at Bierhäuselberg (Vienna region, Austria, 48.13° N, 16.23° E, 350 m AMSL). The site is unmanaged, has an open canopy, and shallow soil. Sampling targeted dominant trees with umbrella-shaped crowns indicating frequent water deficits. Tree rings were dated by standard dendrochronological methods (Speer, 2010).

Generation of the Δ_i' and ε_{met} datasets was described previously (Wieloch *et al.*, 2018, 2022). On average, tree-ring width series started in 1865 (range: 1840 to 1918). To preclude significant isotope shifts due to developmental processes or the contribution of soil-respired CO_2, our isotope analyses start in 1961. At this point, all trees had reached a stable position in the canopy. Since all tree-ring material of each year was combined into annual pools before measuring isotopes, our data represent the species at the site rather than individual trees. To ensure high precision, glucose derivative samples < 20 mg were excluded from measurement (1977, 1978, 1981, and 1982).

Δ_i' was corrected for ^{13}C signal redistribution by heterotrophic triose phosphate cycling (indicated by prime; Wieloch *et al.*, 2018). In this process, part of the sucrose molecules entering tree-ring cells are broken down into the triose phosphates glyceraldehyde 3-phosphate and dihydroxyacetone phosphate. These molecules are in chemical equilibrium which causes ^{13}C signal transfer among carbon positions. Consequently, tree-ring glucose synthesised from triose phosphates inherits a rearranged intramolecular ^{13}C signal distribution. Correcting Δ_i' for heterotrophic triose phosphate cycling restores the original ^{13}C signal distribution.

Notes S2 Hydro-carbon isotope fractionation from 1961 to 1980.

This note investigates whether, during the period 1961 to 1980, Δ_1', Δ_2', and Δ_3' contain systematic variation and whether there is correlated hydrogen and carbon (hydro-carbon) isotope fractionation.

The variance of Δ_1' and Δ_2' during 1961 to 1980 is low compared to 1983 to 1995 (Δ_1': 1.90‰ versus 5.86‰; Δ_2': 1.25‰ versus 5.84‰) and only slightly exceeds the estimated random error variance (Δ_1': 1.12‰; Δ_2': 0.9‰). By contrast, Δ_3' exhibits higher variance during 1961 to 1980 than during 1983 to 1995 (2.43‰ versus 1.34‰) and exceeds the estimated random error variance by about a factor of two (1.06‰). In relation to the total variance, Δ_1', Δ_2', and Δ_3' exhibit 41%, 28%, and 56% systematic variance during 1961 to 1980, respectively.

Furthermore, during 1961 to 1980, Δ_1', Δ_2', and Δ_3' are not significantly correlated (Fig. 4B, Table N1). Similarly, metabolic hydrogen isotope fractionation at glucose H^2, $\varepsilon_{met\ (H2)}$, is neither significantly correlated with Δ_1', Δ_2', and Δ_3' nor with metabolic hydrogen isotope fractionation at glucose H^1, $\varepsilon_{met\ (H1)}$. By contrast,

we found indications for a significant negative correlation between Δ_1' and $\varepsilon_{met\ (H1)}$ (Table N1, r = -0.68, p < 0.01, n = 18). However, looking at the corresponding scatterplot revealed an outlier (Fig. N1, red circle), and removing this outlier from the data removes the correlation between Δ_1' and $\varepsilon_{met\ (H1)}$ (r = -0.39, p > 0.1, n = 17). Thus, there is no evidence for hydro-carbon isotope fractionation during 1961 to 1980.

Table N1 Correlation among isotope series during 1961 to 1980 (n = 18, missing years: 1977, 1978)

	Δ_1'	Δ_2'	Δ_3'	$\varepsilon_{met\ (H1)}$	$\varepsilon_{met\ (H2)}$
Δ_1'	1.00				
Δ_2'	-0.21	1.00			
Δ_3'	-0.25	0.41	1.00		
$\varepsilon_{met\ (H1)}$	-0.68**	0.12	0.19	1.00	
$\varepsilon_{met\ (H2)}$	0.01	0.24	0.09	0.05	1.00

$\varepsilon_{met\ (H1)}$, $\varepsilon_{met\ (H2)}$, Δ_1', Δ_2', and Δ_3' denote hydrogen isotope fractionation caused by metabolic processes at glucose H^1 and H^2, and ^{13}C discrimination at glucose C-1, C-2, and C-3, respectively. Glucose was extracted across an annually resolved tree-ring series of *Pinus nigra* from the Vienna Basin. Significance of series correlation: **, $p \leq 0.01$.

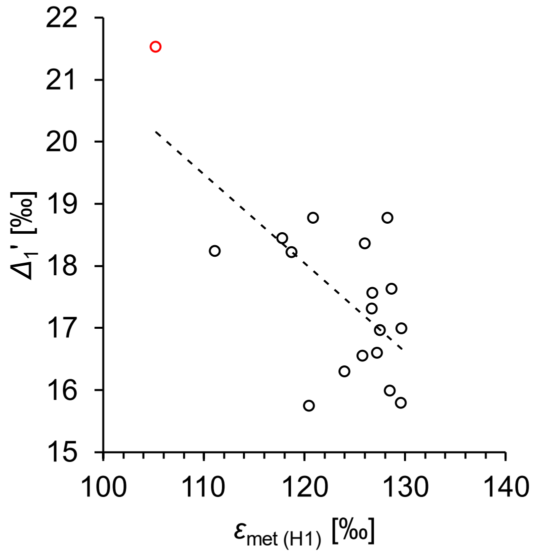

Figure N1 Relationship between metabolic hydrogen isotope fractionation at tree-ring glucose H^1 ($\varepsilon_{met\ (H1)}$) and ^{13}C discrimination at C-1 (Δ_1') during 1961 to 1980. Dashed line, relationship between $\varepsilon_{met\ (H1)}$ and Δ_1'. Red circle, outlier.

Notes S3 Estimated deuterium fractionation due to shifts of the phosphoglucose isomerase reaction.

Table 2 shows reported hydrogen isotope effects of the reaction catalysed by phosphoglucose isomerase (PGI). Full expression of the kinetic isotope effect in the direction glucose 6-phosphate (G6P) to fructose 6-phosphate (F6P) results in 500‰ relative deuterium depletion in F6P as

$$\varepsilon_{\mathrm{inv}} = \frac{1}{\alpha} - 1 = \frac{k_{\mathrm{D}}}{k_{\mathrm{H}}} - 1 = \frac{k_{\mathrm{D}} - k_{\mathrm{H}}}{k_{\mathrm{H}}} = \frac{1}{2} - 1 = -0.5 \qquad \text{(S1)}$$

where $\varepsilon_{\mathrm{inv}}$ denotes deuterium fractionation, α denotes hydrogen isotope effect, and k_{D} and k_{H} denote reaction rate of the heavy and light G6P isotopologue, respectively. By contrast, full expression of the equilibrium isotope effect results in 111‰ relative deuterium enrichment in F6P as

$$\varepsilon_{\mathrm{inv}} = \frac{1}{\alpha} - 1 = \frac{1}{0.9} - 1 = 0.\overline{1} \qquad \text{(S2)}$$

Hence, in the absence of hydrogen exchange with the medium, shifts of the PGI reaction away from equilibrium towards the side of G6P can cause 611‰ deuterium depletion in F6P and 611‰ deuterium enrichment in G6P.

That said, each conversion by PGI was found to be associated with a 0 to 50% probability for hydrogen exchange with the medium (see Fig. 5; Noltmann, 1972). For instance, in spinach leaf extracts, hydrogen transfer in the F6P-to-G6P direction reportedly proceeds with ≈30% of the hydrogen exchanging with the medium, i.e., ≈70% are transferred intramolecularly (Fedtke, 1969). In chlorella extracts, on the other hand, hydrogen transfer occurs 100% intramolecularly, i.e., without exchange with the medium (Simon *et al.*, 1964; Dorrer *et al.*, 1966; Fedtke, 1969). Full intramolecularity may proposedly result if PGI is embedded in a metabolon, a transient multi-enzyme complex (Simon *et al.*, 1964). Additionally, temperature was found to affect the degree of hydrogen exchange with the medium (Rose & O'Connell, 1961; Fedtke, 1969). Taken together, deuterium fractionation by PGI might be dampened by fractional hydrogen incorporation from the medium.

Table S1 Shapiro-Wilk normality test (n = 31).

	Δ_1'	Δ_2'	Δ_3'	Δ_4'	Δ_5'	Δ_6'	Δ	Δ_{1-2}'	Δ_{1-3}'	Δ_{5-6}'	Δ_{4-6}'
W	0.93	0.97	0.99	0.95	0.96	0.94	0.91	0.90	0.90	0.97	0.96
p	**0.04**	0.47	1.00	0.13	0.23	0.06	**0.01**	**0.01**	**0.01**	0.64	0.35

Table S2 F and T test (n = 18 and 13).

	Δ_1'	Δ_2'	Δ_3'	Δ_4'	Δ_5'	Δ_6'	Δ	Δ_{1-2}'	Δ_{1-3}'	Δ_{5-6}'	Δ_{4-6}'
**Variance, 1961-1980**	1.90	1.25	2.43	1.38	3.18	1.86	0.30	0.63	0.60	2.02	1.16
**Variance, 1983-1995**	5.86	5.84	1.34	0.99	2.44	1.89	1.47	4.89	3.25	1.86	1.21
**F test, *p* =**	**0.03**	**0.004**	0.32	0.59	0.68	0.92	**0.003**	**0.001**	**0.002**	0.94	0.88
**Average, 1961-1980**	17.5	7.8	6.4	13.5	19.9	20.4	14.3	12.6	10.5	20.2	17.9
**Average, 1983-1995**	15.8	6.0	6.0	14.2	20.1	19.6	13.6	10.8	9.2	19.9	18.0
**one-tailed T test, *p* =**	**0.02**	**0.01**	0.20	0.06	0.37	0.06	**0.05**	**0.01**	**0.01**	0.28	0.48

Table S3 Pearson's correlations between Δ_i' and ε_{met} series of the period from 1983 to 1995 (n = 13).

	Δ_1'	Δ_2'	Δ_3'	Δ_4'	Δ_5'	Δ_6'
r	-0.59	-0.74	-0.45	-0.05	-0.05	0.07
p	0.03	0.004	0.12	0.87	0.88	0.81

Table S4 Components of variance in Δ_i' series.

	**1983 to 1995**			**1964 to 1995**			
	Δ_1'	Δ_2'	Δ_3'	Δ_4'	Δ_5'	Δ_6'	Δ_{5-6}'
**total variance (‰)**	5.86	5.84	1.34	1.37	2.86	1.76	1.80
**random variance (‰)**	0.71	0.73	0.71	0.85	0.96	0.97	0.48
**fraction of systematic variance (%)**	88	88	47	38	66	45	73

Random variance due to measurement errors in Δ_i' was estimated according to published procedures (Nilsson *et al.*, 1996).

Table S5 Pearson's correlation coefficients and associated levels of significance of ε_{met}-climate relationships for the period from 1983 to 1995 (*n* = 13).

	VPD	*PRE*	*SPEI*$_1$	*SPEI*$_3$	*SPEI*$_4$	*SPEI*$_6$	*SPEI*$_8$	*SPEI*$_{12}$	*SPEI*$_{16}$	*SPEI*$_{24}$	*SPEI*$_{36}$	*SPEI*$_{48}$	*TMP*	*SD*	*RAD*
**MAMJ**	0.55	-0.70	-0.37	-0.17	-0.13	-0.16	-0.20	0.09	0.22	0.07	0.04	0.20	0.15	-0.07	-0.22
**MAMJJ**	0.42	-0.84	-0.43	-0.26	-0.24	-0.24	-0.26	0.00	0.16	0.04	0.00	0.15	-0.04	-0.22	-0.37
**MAMJJA**	0.29	-0.66	-0.27	-0.26	-0.27	-0.27	-0.26	-0.08	0.14	0.04	-0.02	0.11	-0.03	-0.12	-0.31
**MAMJJAS**	0.24	-0.60	-0.20	-0.22	-0.25	-0.29	-0.26	-0.14	0.10	0.03	-0.02	0.08	-0.13	-0.10	-0.31
**MAMJJASO**	0.28	-0.63	-0.24	-0.17	-0.23	-0.29	-0.28	-0.16	0.07	0.02	-0.03	0.06	-0.09	-0.05	-0.31
**MAMJJASON**	0.27	-0.67	-0.27	-0.17	-0.20	-0.29	-0.29	-0.18	0.03	0.02	-0.04	0.03	-0.14	0.01	-0.33
**AMJJ**	0.41	-0.70	-0.41	-0.37	-0.32	-0.26	-0.36	-0.09	0.10	0.01	-0.03	0.12	0.03	-0.17	-0.36
**AMJJA**	0.28	-0.50	-0.28	-0.36	-0.32	-0.28	-0.33	-0.17	0.08	0.01	-0.04	0.08	0.02	-0.07	-0.31
**AMJJAS**	0.23	-0.47	-0.20	-0.31	-0.30	-0.29	-0.31	-0.22	0.05	0.01	-0.04	0.05	-0.11	-0.05	-0.29
**AMJJASO**	0.27	-0.51	-0.24	-0.25	-0.28	-0.29	-0.32	-0.23	0.01	0.00	-0.05	0.03	-0.05	0.00	-0.29
**AMJJASON**	0.26	-0.59	-0.28	-0.24	-0.23	-0.29	-0.32	-0.24	-0.02	0.01	-0.06	0.00	-0.13	0.07	-0.30
**MJJA**	0.22	-0.42	-0.23	-0.40	-0.37	-0.30	-0.33	-0.28	0.01	-0.02	-0.07	0.06	-0.03	-0.06	-0.29
**MJJAS**	0.17	-0.43	-0.17	-0.34	-0.34	-0.30	-0.30	-0.30	-0.02	-0.03	-0.07	0.03	-0.17	-0.03	-0.28
**MJJASO**	0.21	-0.46	-0.21	-0.28	-0.31	-0.30	-0.31	-0.29	-0.06	-0.03	-0.07	0.01	-0.11	0.02	-0.28
**MJJASON**	0.20	-0.53	-0.24	-0.27	-0.26	-0.29	-0.32	-0.29	-0.08	-0.02	-0.07	-0.02	-0.19	0.10	-0.29
**JJAS**	0.08	-0.17	-0.08	-0.31	-0.36	-0.31	-0.28	-0.32	-0.06	-0.02	-0.09	-0.01	-0.41	-0.08	-0.34
**JJASO**	0.13	-0.16	-0.09	-0.23	-0.31	-0.31	-0.29	-0.31	-0.10	-0.03	-0.09	-0.03	-0.40	-0.01	-0.33
**JJASON**	0.13	-0.25	-0.14	-0.22	-0.26	-0.29	-0.29	-0.30	-0.12	-0.01	-0.09	-0.05	-0.40	0.09	-0.35
**JASO**	0.06	0.01	0.07	-0.13	-0.26	-0.31	-0.27	-0.30	-0.13	-0.03	-0.09	-0.07	-0.40	-0.02	-0.32
**JASON**	0.06	-0.09	-0.02	-0.11	-0.19	-0.29	-0.28	-0.29	-0.15	-0.01	-0.09	-0.09	-0.41	0.09	-0.33
**ASON**	0.08	0.10	0.14	0.03	-0.09	-0.27	-0.25	-0.27	-0.15	0.00	-0.08	-0.10	-0.24	0.38	-0.12

Significance levels: ≤ 0.05, light grey; ≤ 0.01, grey; ≤ 0.001, dark grey. Climate parameters: *PRE*, precipitation; *RAD*, global radiation; *SD*, sunshine duration; $SPEI_i$, standardised precipitation-evapotranspiration index of different periods (*i* = 1, 3, 6, 8, 12, 16, 24, 36, 48 months); *TMP*, air temperature; *VPD*, air vapour pressure deficit. Climate data were averaged for all ≥ 4-month periods of the growing season (March to November). Months were abbreviated by their initial letters. ε_{met} denotes hydrogen isotope fractionation caused by metabolic processes at glucose H^1 and H^2. Glucose was extracted across an annually resolved tree-ring series of *Pinus nigra*. Data of the period March to November have been published previously (Wieloch *et al.*, 2022).

Table S6 Linear regression model of $\varepsilon_{met\ (H1)}$ and $\varepsilon_{met\ (H2)}$ as function of March to July precipitation (*PRE*) and growing season air vapour pressure deficit (*VPD*).

$\varepsilon_{met\ (H1)}$ ~ ***PRE* + *VPD*, 1983-1995**			
$R^2 = 0.6$, $adjR^2 = 0.52$, $p = 0.01$, $n = 13$			
	**Estimate**	**± SE**	***p* ≤**
**Intercept**	209	53	0.003
PRE	-0.248	0.074	0.007
VPD	0.0221	0.0694	0.76
$\varepsilon_{met\ (H2)}$ ~ ***PRE* + *VPD*, 1983-1995**			
$R^2 = 0.73$, $adjR^2 = 0.67$, $p < 0.002$, $n = 13$			
	**Estimate**	**± SE**	***p* ≤**
**Intercept**	665	190	0.006
PRE	-1.31	0.27	0.001
VPD	-0.186	0.250	0.47

$\varepsilon_{met\ (H1)}$ and $\varepsilon_{met\ (H2)}$ denote hydrogen isotope fractionation caused by metabolic processes at glucose H^1 and H^2, respectively. Glucose was extracted across an annually resolved tree-ring series of *Pinus nigra* from the Vienna Basin.

References

Abtew W, Melesse AM. **2013**. Chapter 5 - Vapor pressure calculation methods. In: Evaporation and Evapotranspiration. Springer Verlag, 53–62.

Dorrer H-D, Fedtke C, Trebst A. **1966**. Intramolekulare Wasserstoffverschiebung in der Hexosephosphatisomerase-Reaktion bei der photosynthetischen Stärkebildung in Chlorella. *Zeitschrift für Naturforschung* **21b**: 557–562.

Fedtke C. **1969**. Intramolecular hydrogen transfer in isomerisation reactions of sugar phosphates in the Calvin cycle. In: Metzner H, ed. Progress in Photosynthesis Research. Tübingen, 1597–1603.

Klein Tank AMG, Wijngaard JB, Können GP, Böhm R, Demarée G, Gocheva A, Mileta M, Pashiardis S, Hejkrlik L, Kern-Hansen C, *et al.* 2002. Daily dataset of 20th-century surface air temperature and precipitation series for the European Climate Assessment. *International Journal of Climatology* **22**: 1441–1453.

Leal S, Eamus D, Grabner M, Wimmer R, Cherubini P. **2008**. Tree rings of *Pinus nigra* from the Vienna basin region (Austria) show evidence of change in climatic sensitivity in the late 20th century. *Canadian Journal of Forest Research* **38**: 744–759.

Nilsson MB, Dåbakk E, Korsman T, Renberg I. **1996**. Quantifying relationships between near-infrared reflectance spectra of lake sediments and water chemistry. *Environmental Science & Technology* **30**: 2586–2590.

Noltmann EA. **1972**. 9 Aldose-ketose isomerases. In: Boyer PD, ed. The Enzymes. Academic Press, 271–354.

Rose IA, O'Connell EL. **1961**. Intramolecular hydrogen transfer in the phosphoglucose isomerase reaction. *The Journal of Biological Chemistry* **236**: 3086–3092.

Simon H, Dorrer H-D, Trebst A. **1964**. Photosyntheseversuche in Tritiumwasser mit Chlorella. *Zeitschrift für Naturforschung* **19b**: 734–744.

Speer JH. **2010**. *Fundamentals of tree-ring research*. Tucson: The University of Arizona Press.

Wieloch T, Ehlers I, Yu J, Frank D, Grabner M, Gessler A, Schleucher J. **2018**. Intramolecular ^{13}C analysis of tree rings provides multiple plant ecophysiology signals covering decades. *Scientific Reports* **8**: 5048.

Wieloch T, Grabner M, Augusti A, Serk H, Ehlers I, Yu J, Schleucher J. **2022**. Metabolism is a major driver of hydrogen isotope fractionation recorded in tree-ring glucose of *Pinus nigra*. *New Phytologist* **234**: 449–461.
